# Non-oncology drug (meticrane) shows anti-cancer ability in synergy with epigenetic inhibitors and appears to be involved passively in targeting cancer cells

**DOI:** 10.3389/fonc.2023.1157366

**Published:** 2023-05-19

**Authors:** Yulu Wang, Amit Sharma, Fangfang Ge, Peng Chen, Yu Yang, Hongjia Liu, Hongde Liu, Chunxia Zhao, Lovika Mittal, Shailendra Asthana, Ingo G. H. Schmidt-Wolf

**Affiliations:** ^1^ Department of Integrated Oncology, Center for Integrated Oncology (CIO), University Hospital Bonn, Bonn, Germany; ^2^ Department of Neurosurgery, University Hospital Bonn, Bonn, Germany; ^3^ State Key Laboratory of Bioelectronics, School of Biological Science and Medical Engineering, Southeast University, Nanjing, China; ^4^ School of Nursing, Nanchang University, Nanchang, China; ^5^ Translational Health Science and Technology Institute (THSTI), NCR Biotech Science Cluster, Faridabad, Haryana, India

**Keywords:** meticrane, CIK cells, non-oncology drug, epigenetics, cancer

## Abstract

Emerging evidence suggests that chemotherapeutic agents and targeted anticancer drugs have serious side effects on the healthy cells/tissues of the patient. To overcome this, the use of non-oncology drugs as potential cancer therapies has been gaining momentum. Herein, we investigated one non-oncology drug named meticrane (a thiazide diuretic used to treat essential hypertension), which has been reported to indescribably improve the therapeutic efficacy of anti-CTLA4 in mice with AB1 HA tumors. In our hypothesis-driven study, we tested anti-cancer potential meticrane in hematological malignance (leukemia and multiple myeloma) and liver cancer cell lines. Our analysis showed that: 1) Meticrane induced alteration in the cell viability and proliferation in leukemia cells (Jurkat and K562 cells) and liver cancer (SK-hep-1), however, no evidence of apoptosis was detectable. 2) Meticrane showed additive/synergistic effects with epigenetic inhibitors (DNMT1/5AC, HDACs/CUDC-101 and HDAC6/ACY1215). 3) A genome-wide transcriptional analysis showed that meticrane treatment induces changes in the expression of genes associated with non-cancer associated pathways. Of importance, differentially expressed genes showed favorable correlation with the survival-related genes in the cancer genome. 4) We also performed molecular docking analysis and found considerable binding affinity scores of meticrane against PD-L1, TIM-3, CD73, and HDACs. Additionally, we tested its suitability for immunotherapy against cancers, but meticrane showed no response to the cytotoxicity of cytokine-induced killer (CIK) cells. To our knowledge, our study is the first attempt to identify and experimentally confirm the anti-cancer potential of meticrane, being also the first to test the suitability of any non-oncology drug in CIK cell therapy. Beyond that, we have expressed some concerns confronted during testing meticrane that also apply to other non-oncology drugs when considered for future clinical or preclinical purposes. Taken together, meticrane is involved in some anticancer pathways that are passively targeting cancer cells and may be considered as compatible with epigenetic inhibitors.

## Introduction

It has been well established that while anti-cancer/chemotherapy drugs kill cancer cells, they can also damage the healthy cells, causing a plethora of side effects. To avoid this collateral damage, special attention has been paid to the concept of testing non-oncology drugs, prompting the strategy of “drug repurposing,” i.e., drugs already approved for other diseases being identified as potential cancer therapies (1, 2). One of the best examples demonstrating the use of non-oncology drug repurposing is metformin, a classic anti-diabetic drug, that has been under intense investigation across multiple cancer types (3, 4). Of interest is a recent article summarizing several small molecule non-oncology drugs with therapeutic potential in cancer and discussing their putative targets and key pathways relevant to cancer treatment (5).

Notwithstanding all this new progress, it is still too early to definitively assess the success of these proposed potential drugs, although early indications point to positive results. Pushpakom and colleagues recently discussed the challenges being faced by the repurposing community and recommended some innovative ways to address them (6). As a broader concept, the testing of selective (computationally/dockings, high throughput screenings) non-oncology drugs in diverse cancer models, and how they may respond to individual epi(genomic) characteristics remain to be carefully evaluated. In particular, if they can be well combined with other clinically proven drugs/active compounds for cancer. For instance, the combination of epigenetic drugs with chemotherapeutic regimens has proven to be a synergistically relevant as treatment approach (7, 8). More importantly, if the newly selective drug is compatible with cancer immunotherapy related approach.

Considering this, herein, we investigated one non-oncology drug named meticrane (a thiazide diuretic used to treat essential hypertension), which undescribably improved the therapeutic efficacy of anti-CTLA4 in AB1-HA tumor-bearing mice (9). In this hypothesis-driven study, we tested the anti-cancer potential meticrane in hematological malignance (leukemia and multiple myeloma) and liver cancer cell lines. We further extend our analyses by assessing the additive/synergistic potential of meticrane with two epigenetic inhibitors (DNMT1/5AC and HDAC/CUDC-101) in these cells, which was further supported by the molecular docking analysis. Besides, we evaluated the compatibility of meticrane with cytokine-induced killer (CIK) cells, a clinically established effective adoptive immunotherapy approach. To our knowledge, our study is the first attempt to identify and experimentally confirm the anticancer potential of meticrane.

## Materials and methods

### Generation of PBMCs and CIKs

Both Peripheral Blood Mononuclear Cells (PBMCs) and Cytokine-induced killer (CIK) cells were generated, as described previously (10–13). To isolate PBMCs from healthy donors by gradient density centrifugation, Pancoll (Pan-Biotech, Aidenbach, Bavaria, Germany) was used. All donors included in our study were from the blood bank of the University Hospital Bonn. To generate CIK cells, fresh PBMCs were seeded at 3×10^6^ cells/mL in a 75 cm^2^ flask and 1000 U/ml IFN-γ (ImmunoTools GmbH, Aidenbach, Bavaria, Germany) was added after 2 hours. On the following day, 50 ng/ml anti-CD3 antibody (OKT, eBioscience, Thermo Fisher Scientific, Inc. San Diego, CA, USA), 600 U/ml IL-2 (ImmunoTools GmbH, Aidenbach, Bavaria, Germany) and 100 U/ml IL-1β (ImmunoTools GmbH, Aidenbach, Bavaria, Germany) were supplemented. Both PBMCs and CIK cells were cultured in RPMI-1640 medium (Pan-Biotech, Aidenbach, Bavaria, Germany) supplemented with 10% FBS (Sigma-Aldrich Chemie GmbH, Munich, Germany) and 1% penicillin/streptomycin (P/S) (Gibco, Schwerte, Germany), at 37°C, 5% CO_2_, and humidified atmosphere. CIK cells were subcultured every 2-3 days with fresh medium supplemented with 600U/ml IL-2 (1×10^6^ cells/ml). On completion of 14 days of expansion, the CIK cells were collected for the experiments.

### Cell culture, meticrane compound and epigenetic inhibitors

We utilized seven cell lines in this study. The cell lines K562, SK-hep-1, HepG2, and CCD18co were purchased from the American Type Culture Collection (ATCC, Manassas, Virginia, USA). Whereas the cell lines Jurkat, U266 and OPM2 were acquired from the German Collection of Microorganisms and Cell Cultures (DSMZ, Braunschweig, Germany). We cultured K562, U266, Jurkat, and OPM2 in RPMI1640 medium (Pan-Biotech, Aidenbach, Bavaria, Germany) supplemented with 10% FBS (Sigma-Aldrich Chemie GmbH, Munich, Germany) and 1% penicillin/streptomycin (P/S) (Gibco, Schwerte, Germany). While SK-hep-1, HepG2, and CCD18co cells were maintained in EMEM medium (Pan-Biotech, Aidenbach, Bavaria, Germany) supplemented with 10% FBS (Sigma-Aldrich Chemie GmbH, Munich, Germany) and 1% penicillin/streptomycin (P/S) (Gibco, Schwerte, Germany). Meticrane (Sigma-Aldrich Chemie GmbH, Munich, Germany) was dissolved in DMSO and stored at -20°C at a concentration of 200mM. The HDAC inhibitor CUDC-101 (Selleck Chemicals GmbH, Munich, Germany) and the selective HDAC6 inhibitor ACY1215 (Cayman Chemical, Ann Arbor, Michigan, US) was dissolved in DMSO and stored at -20°C at a concentration of 50mM. Also, DNMT1 inhibitor 5-Azacytidine (5AC) (STEMCELL Technologies Germany GmbH, Cologne, Germany) was dissolved in DMSO and stored at -20°C at a concentration of 25mM.

### Cell viability assay and cells number counting assay

In case of suspension cells (K562, U266, OPM2, Jurkat, and PBMCs), the cells were seeded in 96-well flat-bottom plates and then immediately mixed with compounds (meticrane, CUDC-101, 5AC and ACY1215). For adherent cells (SK-hep-1, HepG2, and CCD18co), the drugs were added 4 hours later allowing the cells to adhere first. Considering the different growth rates of tumor cells, 0.5×10^4^ K562 cells, 2×10^4^ U266 cells, 2×10^4^ OPM2 cells, 10×10^4^ PBMCs, 0.5×10^4^ Jurkat cells, 0.25×10^4^ CCD18co cells, 0.3×10^4^ SK-hep-1 cells, and 0.3×10^4^ HepG2 cells were seeded. CCK8 assay (Dojindo EU GmbH, Munich, Germany) was used to determine the cell viability according to its manufacturer’s instructions. In addition, based on the CCK8 results, the combined effects of meticrane and HDAC inhibitors (CUDC101, 5AC and ACY1215) were evaluated using the formula, as described elsewhere (14):


Combination index Q = KE(a+b)/(KEa + KEb - KEa × KEb)

KE represents the killing effect of drugs on cells, while a and b represent drug a and drug b. KE(a+b) means the killing effect of combination drug a and drug b.

According to the combination index Q value, the combined effects of meticrane and epigenetic inhibitors on tumor cells were classified as antagonism (< 0.85), additive (0.85 - 1.15) or synergism (> 1.15). The live cell count was performed using the Canto II flow cytometer (BD Biosciences, Heidelberg, Germany). Hoechst 33258 (Cayman Chemical, Ann Arbor, Michigan, US) was used to stain dead cells, and then precision count beads (BioLegend GmbH, Koblenz, Germany) were used to count the number of live cells.

### Cell proliferation and apoptosis assays

To assess cell proliferation, 0.25μM CFSE (Cell Trace carboxyfl fluorescein succinimidyl ester) (Thermo Fisher Scientific, Eugene, USA) was used to stain 1×10^6^ cells in PBS for 20 minutes at room temperature. While 1ul FITC-annexin (BioLegend GmbH, Koblenz, Germany) and 1ul eBioscience™ 7-AAD Viability Staining Solution (Thermo Fisher Scientific, Eugene, USA) were added to stain tumor cells (100ul volume) for 15mins at room temperature and then were used to assay cell apoptosis. In addition, CellEvent™ Caspase-3/7 Green Flow Cytometry Assay Kit (Thermo Fisher Scientific, Eugene, USA) was used to further evaluate the apoptosis and caspase 3/7 activation level. 0.5μM CellEvent™ Caspase-3/7 Green Detection Reagent and 1μM SYTOX™ AADvanced™ Dead Cell Stain were utilized to stain tumor cells at room temperature for 1h and 5 mins respectively. In these three experiments, 0.5×10^4^ K562 cells, 0.5×10^4^ Jurkat cells and 0.3×10^4^ SK-hep-1 cells were seeded in 96-well flat-bottom plates for 3 days. Of note, adherent cells (SK-hep-1) were added to the wells and meticrane was added 4 hours afterwards. Flow cytometry was performed for these three experiments.

### Cytotoxicity assay of CIK cells

0.25μM CFSE was used to stain tumor cells (1×10^6^) in 1ml PBS, 20 min at room temperature. Subsequently, 1×10^4^ cells of K562 were seeded in 96-well flat-bottom plates and then meticrane and 10×10^4^ CIK cells (4h co-culture time), 10×10^4^ CIK cells (24h co-culture time) and 20×10^4^ CIK cells (24h co-culture time) were added respectively. Likewise, 1×10^4^ SK-hep-1 cells were seeded in 96-well flat-bottom plates and 4 hours later meticrane and 40×10^4^ CIK cells (4h coculture time), 10×10^4^ CIK cells (24h coculture time) and 20×10^4^ CIK cells (24h coculture time) were added. Flow cytometry was used to test the cytotoxicity of CIKs against tumors at 4 and 24 hours of coculture. The cytotoxicity was calculated as following formula: cytotoxicity (%) = ((TC-TT)/TC) ×100. TC: percentage of live tumor cells in control tubes (tumor cells alone), TT: percentage of live tumor cells in test tubes (tumor cells + CIK cells).

### RNA isolation and whole transcriptome analysis

K562 (1×10^5^ cells), Jurakt (1×10^5^ cells) and SK-hep-1 (0.6×10^5^ cells) were seeded in six well plates. As previous described, meticrane was added promptly in K562 and Jurkat cells but in SK-hep-1 cells, it was introduced 4h later. RNA isolation was performed using the RNeasy plus mini kit (QIAGEN GmbH, Hilden, Germany) following the manufacturer’s instructions. Whole transcriptome analysis (3’-mRNA sequencing) was performed at the NGS Core Facility in Bonn, Germany. The data was analyzed using Histat2 (mapping tool) and EdgeR2 (differential analysis tool). The cutoff value (logFC > 2 and FDR< 0.05) was applied to select the differential genes between the untreated and treated meticrane groups. KEGG pathway enrichment analysis (R package: clusterProfiler) were performed on the basis of based on differential genes. The heatmap (R package: pheatmap) was used to show the comparative analysis of differential genes between the untreated and treated meticrane groups.

### Identification of the potential targets of meticrane

To identify potential targets of meticrane, we used previously described methodology (15). Briefly, AML (Acute Myeloid Leukemia) and HCC (Hepatocellular carcinoma) specific gene expression data (log2 (FPKM+1)) (TCGA data from TCGA database, https://portal.gdc.cancer.gov/, project:TCGA-LAML and TCGA-LIHC) and survival data (TCGA data from Ucsc Xena database, https://xenabrowser.net/datapages/, cohort: GDC TCGA Liver Cancer (LIHC) and GDC TCGA Acute Myeloid Leukemia (LAML)) were utilized to imitate the clinical model. Using the TCGA data, we identified genes relevant to survival based on the following criteria: KM curve (*p*< 0.001), Cox regression (*p<* 0.001) and the difference in five-year survival between the low and high gene expression groups of more than 10%. Based on the HR (hazard ratio) value from the Cox regression, we further distinguish between genes with a high risk (poor prognosis) (HR > 1) and those with a low risk (good prognosis) (HR< 1). We then overlap differentially expressed genes (RNA-sequence data) with prognostic genes from TCGA patients’ data. In particular, overlapping of low risk group with up-regulated genes and a high-risk group with down-regulated genes induced by meticrane treatment. All the overlapping genes were used to build protein-protein interaction (string: https://string-db.org/) and KEGG analysis (R package: clusterProfiler).

### Molecular docking and molecular dynamics (MD) simulation

In addition, molecular docking was used to further explore the potential targets of meticrane, particularly focusing on known immune checkpoint (CTLA-4, PD-1, PD-L1, LAG-3, TIM-3, B7-H4, TIGIT, CD73) and epigenetic targets (DNMT1, HDACs). For this purpose, the crystal structures of the corresponding proteins were first extracted from the protein database (www.rcsb.org) and the respective proteins CTLA-4/1I8L, PD-1/4ZQK, PD-L1/6R3K, LAG-3/7TZH, TIM-3/7M3Z, B7-H4/4GOS, TIGIT/5V52, CD73/6TWA, DNA methyltransferase 1/3PTA, and Histone deacetylases (HDAC2/7JS8, HDAC3/4A69, HDAC4/2VQJ, HDAC6/5EDU, HDAC7/3ZNR, HDAC8/7JVU and HDAC10/7U3M were identified. Since for HDACs, three small molecules bound crystal structures were available, therefore, we used all of them to comprehensively analyze different binding modes of ligands in their respective pockets. The protein structures were prepared by using the protein preparation wizard (PPW) module of maestro (Schrodinger LLC, New York, NY, USA) was used to pre-process the structures (16–20). Then, the ligand (meticrane) was prepared using Schrödinger suite (LLC, New York, NY, 2020) LIGPREP (module of maestro), which generates tautomers, and possible ionization states at the pH range 7 ± 2 using Epik (21) and also generates all the stereoisomers of the compound, if necessary (16). The optimization was done using the OPLS3 (Optimized Potentialsfor Liquid Simulations) force field (22). Finally, Glide module of Schrodinger was used to perform the molecular docking and Prime MM-GBSA for binding free energy quantification. The grids were generated using the centroid of co-crystals by using the Receptor Grid Generation panel in Glide. The most favorable ligand-receptor conformations for a drug complex provided by a docking study (18). Glide is a comprehensive and systematic search tool for the molecule of interest from the virtual libraries. The obtained docked poses were then subjected to short MD simulations to study their dynamicity in the pocket. Desmond v3.6 module from Schrodinger suite was used to perform the MD simulations. The systems were built *via* Systems builder using OPLS3 force field and solvated with TIP3P water solvent model. All the complexes were placed in the orthorhombic periodic boundary conditions with a size of repeating buffered units at 10Å. Counter ions were also added to neutralize the systems. An energy minimization step was done for each system for 100ps. The NPT ensemble was employed for the simulations with the Nose-Hover chain thermostat and the martyna-tobias-klein barostat. RESPA integrator was used with a time step of 0.002ps. For short range coulombic interactions, a 9.0 Å cut-off was considered. Bonds to hydrogen were constrained using the MSHAKE algorithm of Desmond. The coordinates were saved at intervals of 10 ps.

### Statistical analysis

All experiments were performed in triplicate and repeated thrice. Besides, the experiments involving CIK cells were performed with three independent donors. FACS data were analyzed using FlowJo V10.6 software (FlowJo, LLC, Ashland, Oregon, U.S.A.). The mean values and standard deviations were used in the figures to demonstrate the experimental data. Also, figures and statistical analyses including one-way or two-way analyses of variance (ANOVA) with Bonferroni’s *post-hoc* test and T-tests were performed using GraphPad Prism v.8.0 (GraphPad Soft-ware, Inc., San Diego, CA, U.S.A.). For bioinformatic data, the statistical analyses and figures were performed by R software. A *p*< 0.05 was considered as significant. *p< 0.05; **p< 0.01; ***p< 0.001; ****p< 0.0001; ns: not significant.

## Results

### Meticrane-induced alteration in the cell viability and proliferation is independent from the apoptosis signaling pathway

**Figure 1:**
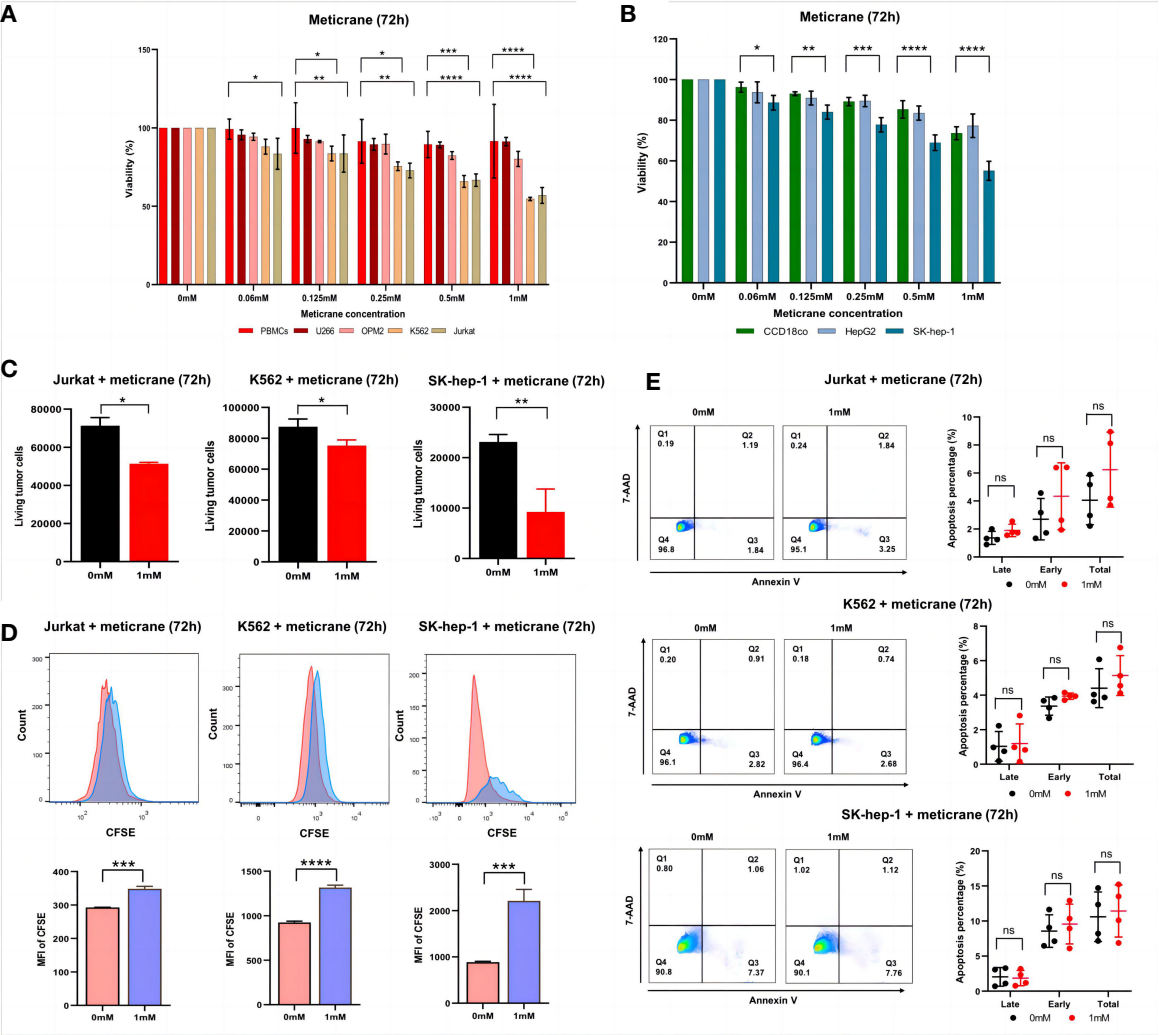
Effect of meticrane on the cell viability, alive cell number, proliferation and apoptosis of tumor cells. **(A)** CCK8 assay for cell viability for leukemia cell lines, myeloma cell lines and control cells. PBMCs (control cells), myeloma (U266 and OPM2) and leukemia (K562 and Jurkat) cells. P value were calculated by two-way ANOVA and Bonferroni’s *post-hoc* test. All data were representative of at least three independent experiments (n≥3). **(B)** CCK8 assay for cell viability for liver cancer cell lines and control cells. CCD18co (control cells), and liver cancer (HepG2 and SK-hep-1) cells. P value were calculated by two-way ANOVA and Bonferroni’s *post-hoc* test. All data were representative of at least three independent experiments (n≥3). **(C)** FASC assay for the relative alive cell number for Jurkat (left), K562 cells (middle) and SK-hep-1 cells (right). All data were representative of three independent experiments (n=3). P value were calculated by T tests. **(D)** Proliferation of Jurkat (left), K562 cells (middle) and SK-hep-1 cells (right). Data are mean ± SD of triplicate measurements; data are one representative of three independent experiments. T test were applied to calculate the p values. MFI, Mean Fluorescent Intensity. **(E)** The apoptosis of K562, Jurkat and SK-hep-1 cells. All data were representative of at four independent experiments (n=4). P value were calculated by two-way ANOVA and Bonferroni’s *post-hoc* test. *p< 0.05, **p< 0.01, ***p< 0.001, ****p< 0.0001, ns, no significant.

To investigate the anticancer effect of meticrane, all cancer cells were co-cultured with meticrane at a concentration of 0.06 to 1 mM at 72 h. The leukemia cells (K562 and Jurkat) were found to be more sensitive to meticrane from 0.125 mM to 1 mM compared to the control cells (PBMCs) (**Figure 1A**). The cell viability was found to be decrease with increase in meticrane concentration in K562 (0.06mM: *p*=0.2384, 0.125mM: *p*=0.0264, 0.25mM: *p*=0.0323, 0.5mM: *p*=0.0005, 1mM: *p*<0.0001) and Jurkat (0.06mM: *p*=0.0103, 0.125mM: *p*=0.0073, 0.25mM: *p*=0.0017, 0.5mM: *p*<0.0001, 1mM: *p*<0.0001). However, myeloma cells (U266 and OPM2) (**Figure 1A**) showed no significant difference at any concentration compared to the controls (all *p* values at each concentration were more than 0.05). Likewise, in liver cells, SK-hep-1 cells showed significantly lower viability compared to the control cells (CCD18co cells), whereas HepG2 cells showed no significant difference (**Figure 1B**). The cell viability was found to be decrease with increase in meticrane concentration in SK-hep-1 (0.06mM: *p*=0.011, 0.125mM: *p*=0.0025, 0.25mM: *p*=0.0001, 0.5mM: *p*<0.0001, 1 mM: *p*<0.0001) and HepG2 (all *p* values at each concentration were more than 0.05) (**Figure 1B**). Considering cell viability is directly correlated to the viable/alive cells, we next investigated and found that the number of alive K562 cells (*p*=0.026), Jurkat cells (*p*=0.0013), and SK-hep-1 cells (*p*=0.0011) significantly decreased in the meticrane (1mM)-treated group compared with the untreated group after 72 h (**Figure 1C**), suggesting that meticrane could reduce the number of tumor cells. In addition, the MFI (Mean fluorescent intensity) of CFSE (Cell Trace carboxyfl fluorescein succinimidyl ester) of K562 cells (*p*<0.0001), Jurkat cells (*p*=0.0002), and SK-hep-1 cells (*p*=0.0007) was also found to be higher in the presence of meticrane (**Figure 1D**), suggesting that the proliferation of these cell were inhibited due to meticrane. Interestingly, no significant difference was observed between early and late apoptosis in all observed groups of K562 cells, Jurkat cells and SK-hep-1 cells by using Annexine V and 7AAD dyes (**Figure 1E**). Besides, we checked both apoptosis and caspase 3/7 activation level potentially caused by meticrane, and found no alterations by using CellEvent™ Caspase-3/7 Green Flow Cytometry Assay Kit (**Supplementary Figure 1**). Both two apoptosis experiments suggested that the strongly reduced cell viability is independent of the apoptosis-related signaling pathway. It can therefore be concluded that meticrane may induce the alteration of cell viability and proliferation in selected hematologic and liver cancer cells, through independent of the apoptosis signaling pathway.

### Meticrane showed additive/synergistic effect with epigenetic inhibitors

**Figure 2:**
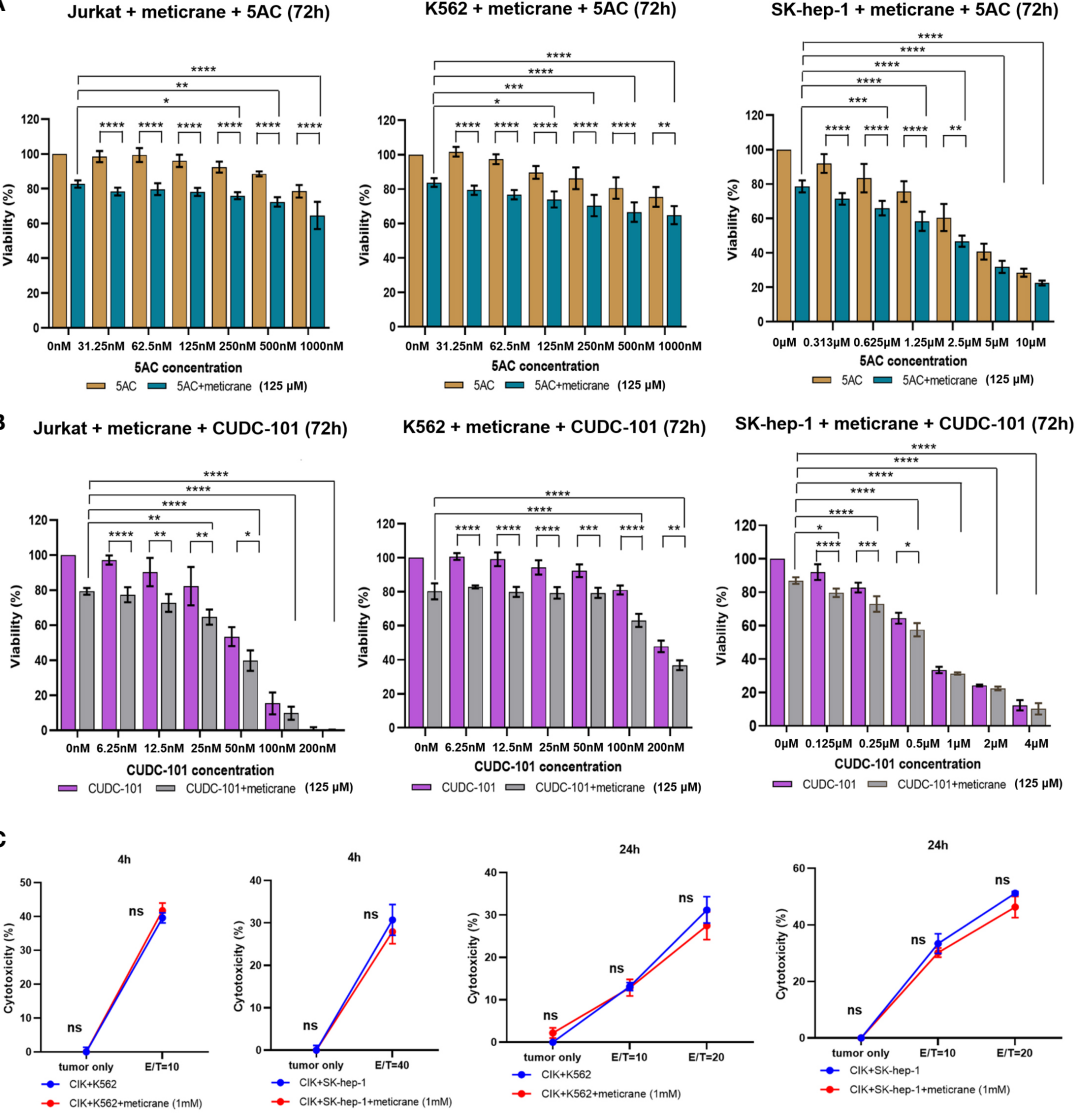
The combination effect of meticrane with epigenetic inhibitors or CIK cells. 5AC **(A)** or CUDC-101 **(B)** were used to test the cell viability (CCK8 assay) in Jurkat, K562 and SK-hep-1 cells. All data were representative of at least three independent experiments (n≥3). When comparing these two groups (no meticrane group vs. combined meticrane group), p-values were calculated using two-way ANOVA and Bonferroni’s *post-hoc* test. When comparing the different dose in the group with meticrane, the p-value was calculated using a one-way ANOVA and the Bonferroni *post-hoc* test. **(C)** Cytotoxicity of CIK cells with/without meticrane against K562 and SK-hep-1 cells at 4 hours (left) and 24 hours (right) point time. Data are mean ± SD of triplicate measurements; data are one representative of three independent experiments. T test (4h) and two-way ANOVA (Bonferroni’s *post-hoc* test) (24h) were applied to calculate the *p* values. *p<0.05, **p<0.01, ***p<0.001, ****p<0.0001. ns, no significant.

Whether the effects of meticrane led to the alteration in cell viability and proliferation in leukemia cells (K562 and Jurkat) and liver cancer cells (SK-hep-1) can be enhanced with known epigenetic inhibitors, we assayed both the DNMT1 inhibitor (5AC) and HDAC inhibitor (CUDC-101) in these cells for 72 h using CCK8 assay (**Figures 2A, B**). To ensure consistency, meticrane (125μM) was combined with 5AC (31.25nM-1000nM) and CUDC -101 (6.25nM-200nM) against K562 and Jurkat cells, whereas CUDC -101 (0.125μM-4μM) or 5AC (0.313μM-10μM) was optimized against SK-hep-1 cells. Of interest, in all cell lines, the addition of 5AC in combination with meticrane showed significant differences in Jurkat cells (all *p*<0.0001), K562 cells (1000nm: *p*=0.0033, 31.25-500nM: all *p* values< 0.0001) and SK-hep-1 cells (0.313-1.25μM: all *p*<0.05, 2.5μM: *p*=0.0014) compared to the 5AC alone. Notably, in Jurkat cells, meticrane (125μM) in combination with 5AC (250nM: *p*=0.0499, 500nM: *p*=0.001 and 1000nM: *p*<0.0001) showed higher inhibitory effect than meticrane alone (**Figure 2A**). This effect was also observed in K562 (125nM: *p*=0.0104, 250nM: *p*=0.0004, 500nM: *p*<0.0001 and 1000nM: *p*<0.0001) and SK-hep-1 cells (0.625μM: *p*=0.0006, 1.25μM-10μM: all *p*<0.0001). Like 5AC, CUDC -101 also in combination with meticrane showed significant differences in Jurkat cells (6.25nM: *p*=0.0005, 12.5nM: *p*=0.0019, 25nM: *p*=0.0018, 50nM: *p*=0.0221), K562 cells (6.25nM-25nM: all *p*<0.0001, 50nM: *p*=0.0002, 100nM: *p*<0.0001, 200nM: *p*=0.0016), and SK-hep-1 cells (0.125μM: *p*<0.0001, 0.25μM: *p*=0.0001, 0.5μM: *p*=0.0116) compared to the CUDC-101 alone. The higher inhibitory effect of meticrane in combination with CUDC-101 was observed in Jurkat cells (25nM: *p*=0.0033, 50nM-200nM: all *p*<0.0001), K562 (100nM: *p*<0.0001, 200nM: *p*<0.0001) and Sk-hep-1 cells (0.125μM: *p*=0.0126, 0.25μM-4μM: *p*<0.0001) compared to meticrane alone. We also calculated the combination index Q values of meticrane with different concentrations of CUDC101 or 5AC on tumor cells (K562, Jurkat and SK-hep-1), and found mainly the additive/synergetic effects (**Tables 1**, **2**).

**Table 1:** Combination index Q of meticrane with CUDC101 in K562, Jurkat and SK-hep-1 cells.

Jurkat			K562			SK-hep-1		
meticrane	CUDC101	Index Q	meticrane	CUDC101	Index Q	meticrane	CUDC101	Index Q
125μM	0nM	1.00	125μM	0nM	1.00	125μM	0μM	1.00
125μM	6.25nM	0.99	125μM	6.25nM	0.90	125μM	0.125μM	1.02
125μM	12.5nM	0.96	125μM	12.5nM	0.98	125μM	0.25μM	0.96
125μM	25nM	1.02	125μM	25nM	0.85	125μM	0.5μM	0.96
125μM	50nM	1.05	125μM	50nM	0.80	125μM	1μM	0.97
125μM	100nM	1.03	125μM	100nM	1.05	125μM	2μM	0.98
125μM	200nM	1.00	125μM	200nM	1.03	125μM	4μM	1.01

**Table 2:** Combination index Q of meticrane with 5AC in K562, Jurkat and SK-hep-1 cells.

Jurkat			K562			SK-hep-1		
meticrane	5AC	Index Q	meticrane	5AC	Index Q	meticrane	5AC	Index Q
125μM	0nM	1.00	125μM	0nM	1.00	125μM	0μM	1.00
125μM	31.25nM	1.17	125μM	31.25nM	1.39	125μM	0.313μM	1.03
125μM	62.5nM	1.14	125μM	62.5nM	1.26	125μM	0.625μM	0.99
125μM	125nM	1.06	125μM	125nM	1.05	125μM	1.25μM	1.03
125μM	250nM	1.02	125μM	250nM	1.07	125μM	2.5μM	1.02
125μM	500nM	1.03	125μM	500nM	1.03	125μM	5μM	1.00
125μM	1000nM	1.01	125μM	1000nM	0.95	125μM	10μM	1.00

### Meticrane showed no compatibility with cytokine-induced killer cells

To further investigate the potential effect of meticrane with immunotherapy, cytokine-induced killer cells (CIKs) were assessed with meticrane. Meticrane (1mM) in combination with CIK cells was tested against K562 cells and SK-hep-1 cells. In particular, meticrane did not change the cytotoxicity of CIKs against K562 cells (*p*=0.2391) and SK-hep-1 cells (*p*=0.424) tested at time point 4h (**Figure 2C**). Due to this different sensitivity of CIKs against K562 and SK-hep-1 cells at 4h, we applied a different E/T ratio for K562 (E/T=10) and SK-hep-1 (E/T=40). Likewise, meticrane did not change the cytotoxicity of CIKs against K562 cells (E/T=10 *p*=1, E/T=20 *p*=0.1548) and SK-hep-1 cells (E/T=10 *p*=0.344, E/T=20 *p*=0.0673) tested at time point 24h (**Figure 2C**). Of note, as shown in the tumor only group in **Figure 2C** at 4h and 24 time point, meticrane alone (without CIKs) did not show cytotoxicity against K562 (4 hours *p*=1, 24 hours *p*=0.6757) or SK-hep-1 cells (4 hours *p*=1, 24 hours *p*=1) at either 4 hours or 24 hours (**Figure 2C**). Overall, meticrane showed no compatibility with cytokine-induced killer cells.

### Meticrane exerts no effect on cancer-associated signaling pathways in cancer cells

**Figure 3:**
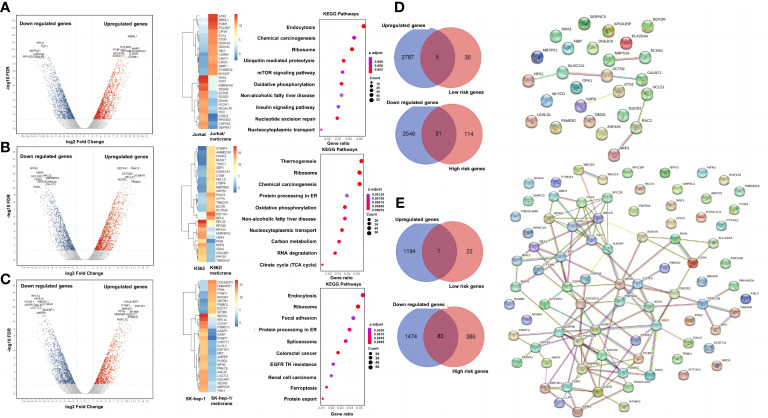
A genome wide transcriptional analysis and correlation with the patient survival. The differentially expressed genes, heat map of the 30 most important differential genes and KEGG pathways comparing the meticrane-treated group and the meticrane-untreated group (DMSO control group) in Jurkat **(A)**, K562 **(B)** and SK-hep-1 **(C)**. Venn diagram (left) of meticrane upregulated/downregulated genes and low/high risk genes and protein-protein interaction (right) of overlapping genes between upregulated/downregulated genes and low/high risk genes for leukemia **(D)** and liver cancer **(E)**.

A genome-wide transcriptional analysis was performed to investigate the transcriptional changes in the cells treated with meticrane (**Figures 3A-C**). Based on differential genes between untreated and treated meticrane groups, we obtained meticrane induced significantly upregulated/downregulated genes from leukemia cell lines (Jurkat: 1500 up-regulated and 1519 down-regulated, **Supplementary Table 1**; K562: 1521 up-regulated and 1237 down-regulated, **Supplementary Table 2**) and liver cancer cell line (SK-hep-1: 1195 up-regulated and 1557 down-regulated, **Supplementary Table 3**). Using KEGG enrichment analysis to identify the ten most enriched metabolic pathways, we found that the leukaemia cell lines (Jurkat and K562) were highly enriched in oxidative phosphorylation, mTOR signalling, RNA degradation and regulation of cancer-related metabolic pathways. For the liver cancer cell line (SK-hep-1), there was significant enrichment in ferroptosis, focal adhesion and signaling pathways that play an important role in cancer regulation, such as protein processing in the ribosome and endoplasmic reticulum. Thus, meticrane showed no direct/predominant effect on cancer-related signaling pathways in leukemia cell lines, and a distant impact (i.e., pathways not directly involved in cancer) to cancer in liver cancer cells.

### Meticrane induced differentially expressed genes showed association with survival-related genes in cancer

We identified survival relevant genes for AML (high risk genes: n=135 and low risk genes: n=35; **Supplementary Table 4**) and HCC (high risk genes: n=469 and low risk genes: n=23; **Supplementary Table 5**) were found using TCGA datasets. Subsequently, the low-risk genes were correlated with the up-regulated genes induced by meticrane (RNA-sequence) and the high-risk genes were correlated with the down-regulated genes induced by meticrane. In this pattern, we identified groups of overlapping genes in for AML (low-risk/up-regulated genes: n=5; high-risk/down-regulated genes: n=21) and HCC (low-risk/up-regulated genes: n=1; high-risk/down-regulated genes: n=83) (**Figures 3D, E**; **Supplementary Table 6**). By combining our *in vitro* data and information from TCGA’s publicly available clinical portal, we described 110 genes (AML=26 genes; HCC=84 genes) (**Supplementary Table 6**) as potential targets of meticrane in these two cancers. We then established PPI (protein-protein interaction, cutoff interaction value: 0.4.) on these genes and found moderate to weak interactions in HCC and AML, respectively (**Figures 3D, E**). Using KEGG analysis of these selective genes, we also found that they are specifically involved in non-cancer pathways (**Supplementary Figure 2**).

### Molecular docking and molecular dynamics (MD) simulation analysis confirmed the binding affinity of meticrane with known oncological targets

**Figure 4:**
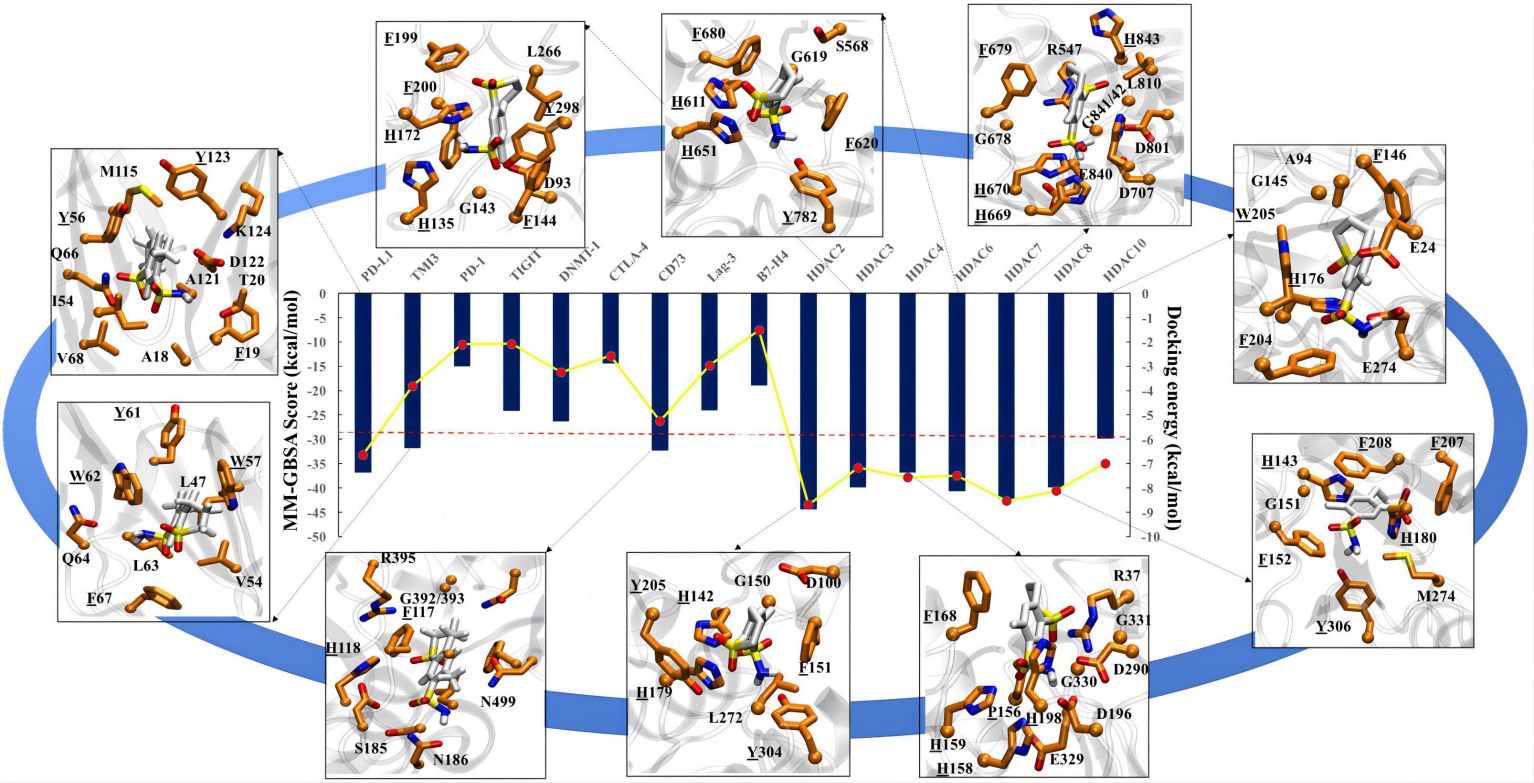
Molecular docking analysis for meticrane. Molecular docking of meticrane on established oncological targets is shown. The bi-axis docking energy and MM-GBSA scores (in kcal/mol) are marked. The cut-off is shown in a red dotted line. The interaction mapping of all targets with significant docking energy and MM-GBSA scores (>= to cut-off) are highlighted. In the interaction map, the meticrane and amino acids of each protein are shown in licorice color and colored by atoms as C: white/orange, O: red, N: blue, S: yellow, respectively. From the interaction map the aromatic residues that appear to be essential for the binding and stability of the metachrane have been identified (highlighted with underlining).

To further explore the potential targets of meticrane, we performed a molecular docking analysis by aligning Meticrane against known immune checkpoints (CTLA-4, PD-1, PD-L1, LAG-3, TIM-3, B7-H4, TIGIT, CD73) and epigenetic targets (DNMT1, HDACs) (**Figure 4**; **Supplementary Figure 3**). On the basis of molecular docking followed by MM-GBSA scores, it is evident that meticrane has considerable binding affinity against some oncological targets such as PD-L1, TIM-3, CD73, and HDACs (HDAC2, HDAC3, HDAC4, HDAC6, HDAC7, HDAC8 and HDAC10) (**Figure 4**). Given the small size of meticrane, the binding affinity score is considerable, suggesting that these proteins may be possible targets. As proof of principle, we selected HDAC6 for further analysis. Interestingly, when HDAC6 inhibitor (ACY1215) was combined with meticrane, a significantly high impact on the viability of tumor cells (K562, Jurkat and SK-hep-1) were observed (**Supplementary Figures 4A-C**). Additionally, we found that meticrane with ACY1215 has additive/synergistic effects against tumor cells, based on the combination index Q values (**Supplementary Figure 4D**).

To extend the analysis, we also performed MD simulations and investigated the dynamic behavior of the protein and ligands using the RMSD parameter, in which the structural deviations in the molecule are calculated over time with respect to the initial structure (docked pose). The RMSD of the ligands (plateau reached) confirms the stability of the meticran in the pocket of each protein, suggesting that these proteins may be of interest as potential targets for thorough experimental validation in the future (**Supplemental Figure 5**).

## Discussion

Certainly, there are enormous number of chemotherapeutic agents and targeted anti-cancer drugs, however, their side effects on the patient’s healthy cells/tissues are not negligible. Given that the development of new anti-tumor drugs requires extensive preclinical and clinical studies, drug repositioning (also known as “drug repurposing”) has emerged as a rapid alternative strategy, particularly related to non-oncology drugs (23). Moreover, several putative non-oncology drugs have been predicted, but their potential as future cancer therapeutics is unknown (24). Broadly, metformin is currently a typical example of a non-oncology anticancer drug (25), driven by the hypothesis of reducing the availability of glucose and insulin to slow down the tumor growth and progression. Herein, we tested another non-oncological drug named as meticrane, a thiazide diuretic commonly used to treat essential hypertension. Previously, meticrane in combination with CTLA-4 treatment was reported to improve the survival of mesothelioma mice (9), however, the anticancer effect of meticrane in tumors remained unexplored. In the current study, for the first time, we investigated the anti-cancer ability of meticrane in hematologic malignancies (myeloma and leukemia) and liver cancer cell lines.

We first cultured meticrane with cancer cells and found that leukemia cells (K562 and Jurkat) were more sensitive, whereas myeloma cells (U266 and OPM2) lacked a similar response. Similarly, some liver cancer cells (SK-hep-1) responded more effectively to meticrane, whereas others did not (HepG2). Notably, all the cell lines included in this study have a very distinctive (epi-)genetic profile, e.g., K562 (adult female/53 years, TP53 mutation), Jurkat (young male/14 years, TP53, BAX, NOTCH1, MSH1/6, INPP5D mutations), U266 (adult male/53 years, TP53, BRAF, TRAF3, MSH6 mutations), OPM2 (adult female/56 years, TP53, SMAD2, CDKN2A, FGFR3 mutations), SK-hep-1 (adult male/52 years, BRAF, CDKN2A mutations), and HepG2 (young male/15 years, TERT, NRAS mutations). Thus, we confirmed that meticrane indeed has an anti-cancer potential that specifically targets certain genetic constellations. Certainly, some discrepancies in the experiments are expected owing to heterogeneity among cancer cell lines in addition to (epi-)genomic factors (26). In addition, we also tested and confirmed that meticrane has the potential to significantly reduce the number of tumor cells and proliferation. Particularly, these effects were validated in three cell lines (K562, Jurakt and SK-hep-1). We also examined whether apoptosis-related signaling pathways (cell death) might contribute to this noticeable cytotoxic effect, but confirmed that no evidence of apoptosis was detectable in K562, Jurkat, or SK-hep-1 cells, suggesting that it may inhibit cancer cell proliferation in an apoptosis-independent manner. In fact, some previous evidence suggests that a few compounds can cause cancer cell death *via* an apoptosis-independent pathway (27, 28). Whether meticrane would be of greater benefit to patients, who do not respond to clinical drugs due to apoptosis resistance, will be of future interest.

Next, we combined meticrane with the established epigenetic inhibitors CUCD-101 (HDACi) and 5AC (DNMTi), as epigenetic alterations are also known to influence numerous aspects of cancer and such inhibitors have already been tested in multiple cancer/clinical studies (29). Noticeably, meticrane in combination with CUDC-101 or 5AC showed a higher inhibitory effect in hematological malignancies (K562 and Jurkat cells) and in liver cancer (SK-hep-1) cells compared to meticrane or epigenetic inhibitors alone. The combination of meticrane and epigenetic inhibitors (CUDC-101 or 5AC) showed additive/synergistic effects on K562, Jurkat and SK-hep-1 cells. Therefore, this combo (meticrane+epigenetic inhibitors) might be a possible replacement for toxic substances used for cancer treatment, however, *in-vivo* studies are warranted in this context. Motivated by the optimistic results attained with a cocktail of meticrane and epigenetic inhibitors for anticancer efficacy, we subsequently tested its suitability for immunotherapy against cancers, in particular, cytokine-induced killer (CIK) cell therapy. Being a pioneer of CIK cell therapy (30), we have already demonstrated the favorable effect of CIK cells with known cancer inhibitors (e.g. PD-1/PD-L1) (31) and even epigenetic compounds (e.g. HDAC) (32). Intriguingly, meticrane showed no response to the cytotoxicity of CIKs against K562 cells and Sk-hep-1 cells over 4-24 hours of treatment. At this point, we cannot conclude whether similar effect will also prevail for other immunomodulatory effects of CIK cells when used under *in vivo* conditions. To our knowledge, this is the very first study to test any non-oncology drug against CIK cells. To gain better insight into the transcriptional role of meticrane, we performed genome-wide transcriptional analyses in both untreated and treated groups of meticrane in Jurkat, K562 and SK-hep-1 cells. Interestingly, we identified both up-regulated and down-regulated genes in all experimental groups, showed no direct/predominant effect on cancer-related signaling pathways in leukemia cell lines, and a distant impact to cancer in liver cancer cells. This suggests that meticrane can induce changes in cancer cells (as confirmed by the changes in cell viability and proliferation), but in a passive manner. As a proof of concept, we also overlap the obtained meticrane induced differentially expressed genes with the cancer specific survival data from the publicly available TCGA dataset and found a correlation among them. Therefore, it is reasonable to speculate that meticrane is involved in some anticancer pathways that are passively involved in targeting cancer cells and may be considered as compatible with other clinically safe drugs, particularly epigenetic inhibitors. These findings also prompted us to conduct molecular docking analysis in order to further explore the potential targets of meticrane. We specifically focused on known immune checkpoints (CTLA-4, PD-1, PD-L1, LAG-3, TIM-3, B7-H4, TIGIT, CD73) and epigenetic targets (DNMT1, HDACs). Of interest, we found considerable binding affinity scores of meticrane against PD-L1, TIM-3, CD73, and HDACs. To validate, we focused on HDAC6 for further analysis, and found a significantly high impact on the viability of tumor cells when HDAC6 inhibitor (ACY1215) was combined with the meticrane. Since meticrane showed additive/synergistic effects with CUDC101, 5AC and ACY1215 in our analysis, this could partly explain its positive molecular binding affinity with these epigenetic target proteins. Certainly, additional analyses for other putative targets are warranted. On a broader view, it is reasonable to speculate that meticrane may not alter any specific cancer-related pathway, but may exert its distant effects on the cancer cells (passively) *via* well-known immune-regulatory/epigenetic signaling pathways, preferably *via* targeting PD-L1, TIM-3, CD73, and HDACs.

It is equally important to address the limitations and future prospects of our (similar) studies, for instance, 1) As we have observed in case of meticrane, other non-oncology drugs may also not have direct targets associated with cancer, and therefore experiments like RNA sequencing (whole transcriptome analysis) studies following co-cultures in cancer cells may not be sufficient to draw any conclusions. 2) It is entirely possible that these drugs show anticancer activity only at high doses, so screening with variable concentrations (min to max) is recommended. At least in the case of meticrane, synthesis of other next-generation compounds (based on its structure) with a stronger tendency to inhibit the proliferation of cancer cells may solve this problem to some extent. 3) The genetic/epigenetic background of the cancer type and even gender differences may lead to different outcomes with these drugs in clinics. Specifically, when it is also known about the considerable overlapping between gene expression variation and the association of altered mutational pathways across the cancer genome (33, 34). Therefore, larger panels of cancer cell lines with multiple genetic constellations are necessary to confirm their potential mode of action. 4) Considering that cancer patients have a limited therapeutic window, it will be a significant question to follow whether non-oncology drugs (presumably alone) are sufficient to prolong the survival, especially in patients without any signs of cancer for a certain period of time after the treatment. 5) Such drugs may not be appropriate for all cancer immunotherapy types, hence, a critical selection of specific immunotherapy (broadly activating the immune system and/or precisely targets of the tumor) should be pre-addressed. Overall, we were able to show that meticrane, a non-oncology drug, exhibits anticancer potential with epigenetic inhibitors *in-vitro*, but not with cytokine-induced killer (CIK) cells.

## Conclusions

Non-oncology drug (meticrane) effectively synergizes with epigenetic inhibitors in leukemia and liver cancer cells. Though we have demonstrated its anticancer ability, its mechanistic inference is still unclear. In the current study, we also expressed some important concerns encountered during the meticrane testing, which are also relevant to other non-oncology drugs when considering their future clinical or preclinical use.

## Data availability statement

The data supporting the findings of this study are available from the corresponding author [I.G.H.S-W] on request.

## Author contributions

Conceptualization, YW, AS, and IS-W; methodology, YW, YY, HJL, HDL, LM, SA, and CZ; validation, YW, FG and PC; writing—original draft preparation, YW, AS, and IS-W, project administration, AS and IS-W; funding acquisition, IS-W. All authors contributed to the article and approved the submitted version.

## Supplementary material

Supplementary Figure 1 Evaluating apoptosis and Caspase3/7 activation by using CellEvent™ Caspase-3/7 Green Flow Cytometry Assay Kit

Supplementary Figure 2 KEGG enrichment analysis of overlapping genes found between meticrane induced differentially expressed genes and survival-related genes in cancer.

Supplementary Figure 3 Molecular docking analysis.

Supplementary Figure 4 The combination effect of meticrane with ACY1215 (selective HDAC6 inhibitor). The viability of Jurkat (A), K562 (B) and SK-hep-1 (C) cells in presence of ACY1215 with/without meticrane. D) Combination index Q of Meticrane and ACY1215 on K562, Jurkat and SK-hep-1 cells.

Supplementary Figure 5 Molecular dynamics

Supplementary Table 1 Meticrane induced differentially expressed genes in Jurkat.

Supplementary Table 2 Meticrane induced differentially expressed genes in K562.

Supplementary Table 3 Meticrane induced differentially expressed genes in SK-hep-1.

Supplementary Table 4 Survival-related genes in AML.

Supplementary Table 5 Survival-related genes in HCC.

Supplementary Table 6 Overlapping genes between meticrane induced differentially expressed genes and survival-related genes in cancers.
