# Supplementary figures and images for: Non-oncology drug (meticrane) shows anti-cancer ability in synergy with epigenetic inhibitors and appears to be involved passively in targeting cancer cells
Source: Front Oncol. 2023 May 19;13:1157366. doi: 10.3389/fonc.2023.1157366 (PMC10235775; doi:10.3389/fonc.2023.1157366)

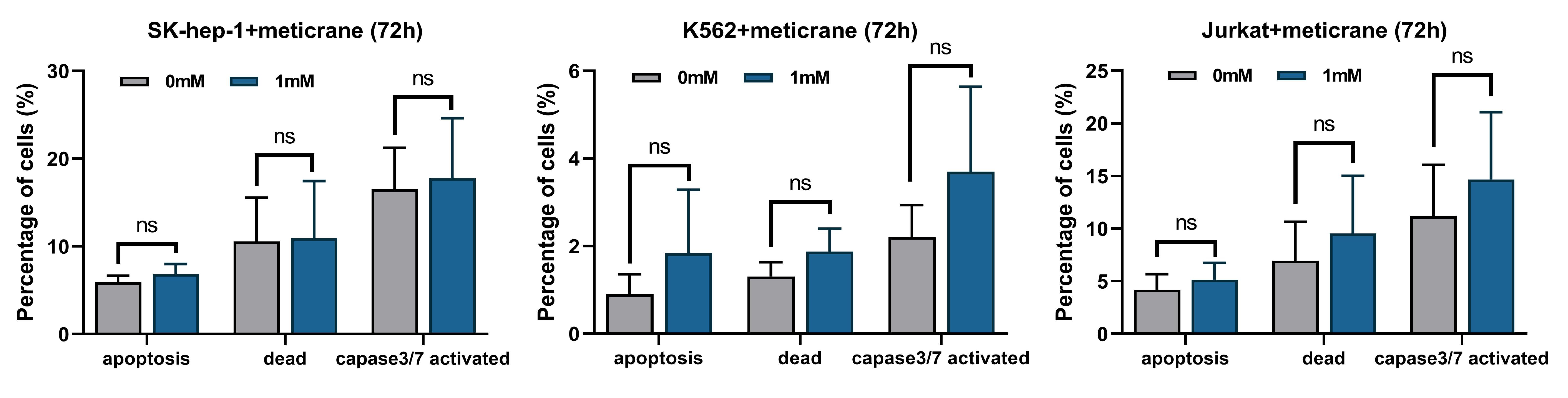

Supplement: Supplementary Figure 1 — Evaluating apoptosis and Caspase3/7 activation by using CellEvent™ Caspase-3/7 Green Flow Cytometry Assay Kit [file Image_1.jpeg]

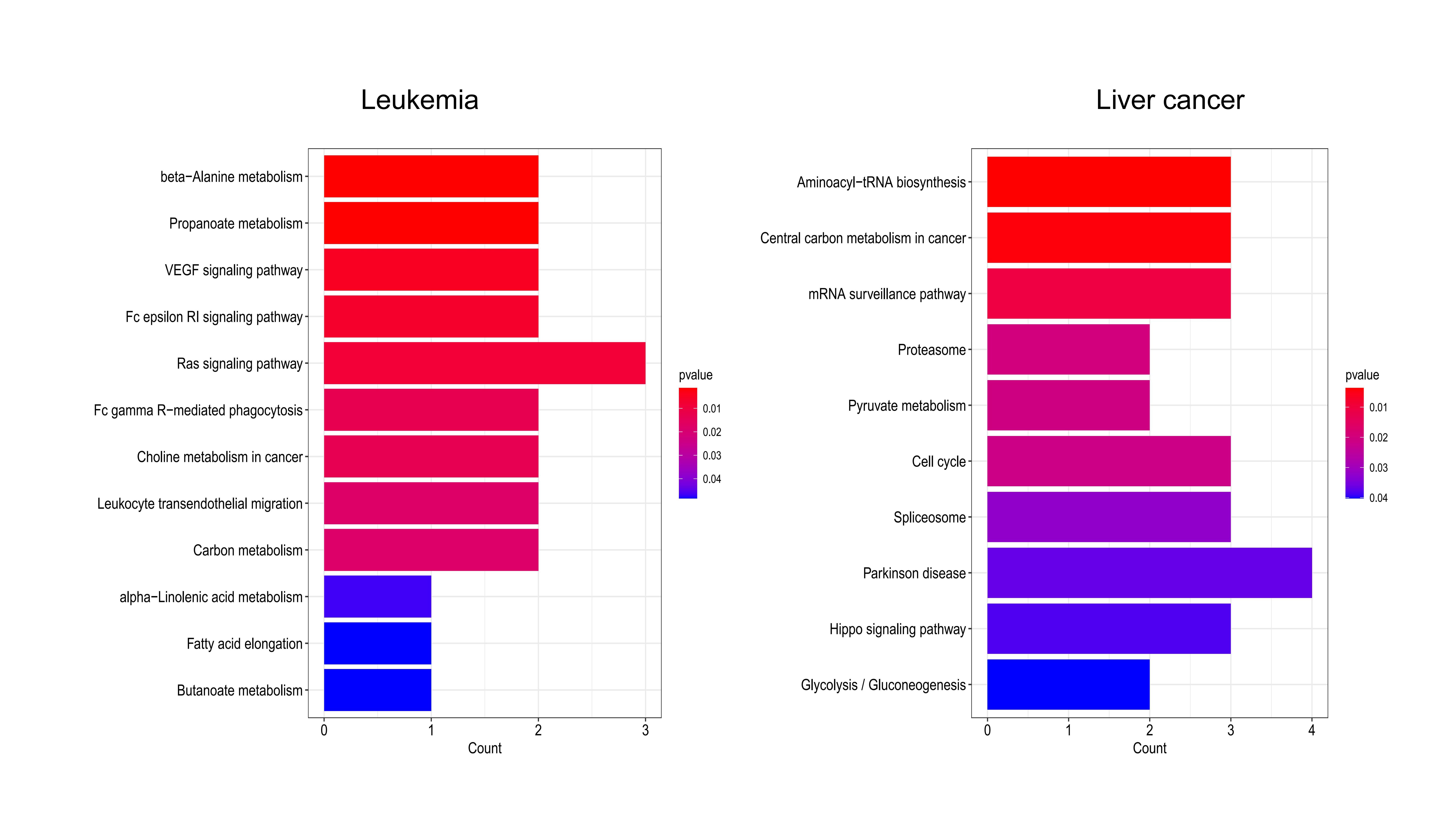

Supplement: Supplementary Figure 2 — KEGG enrichment analysis of overlapping genes found between meticrane induced differentially expressed genes and survival-related genes in cancer. [file Image_2.jpeg]

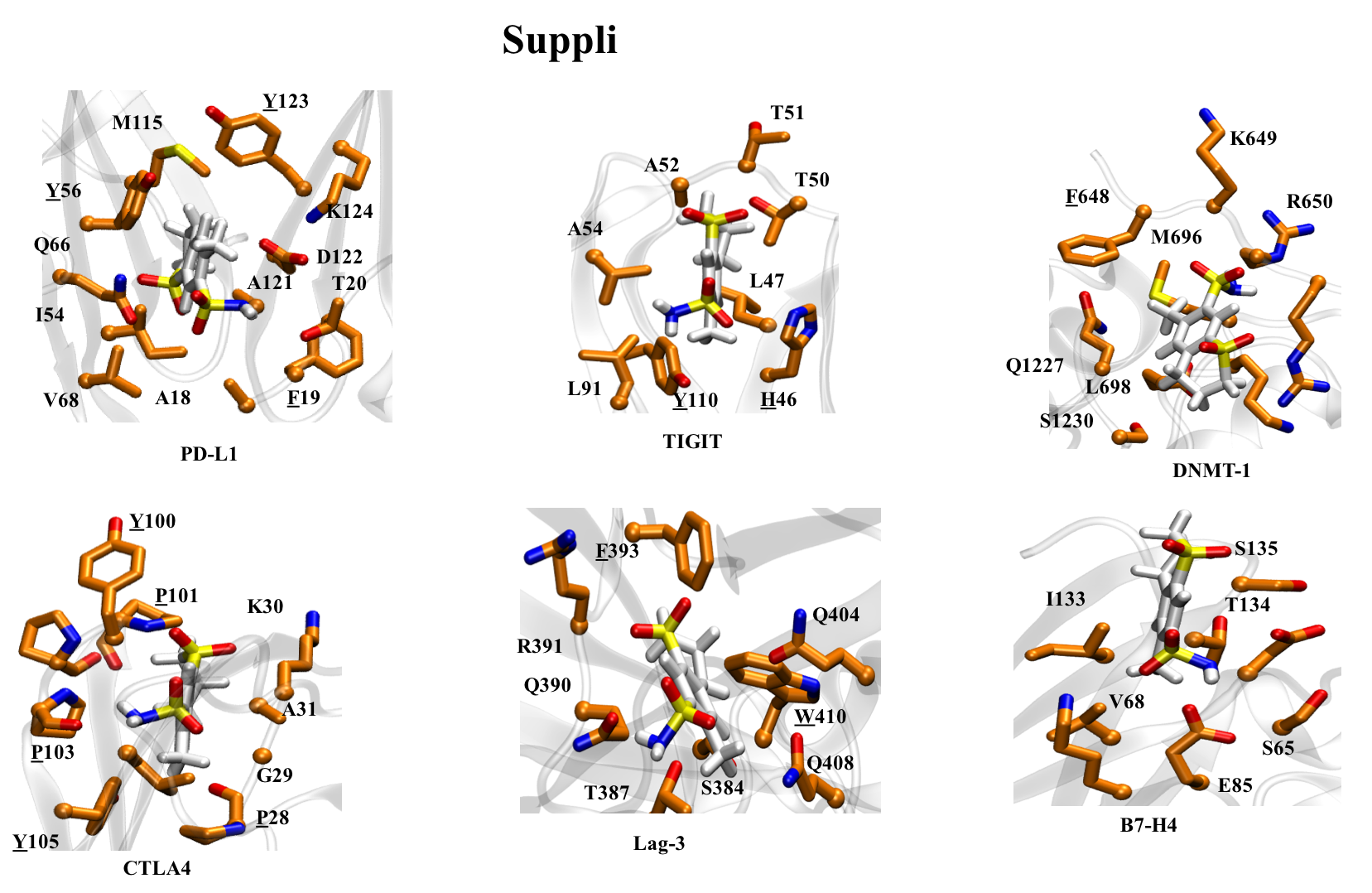

Supplement: Supplementary Figure 3 — Molecular docking analysis. [file Image_3.png]

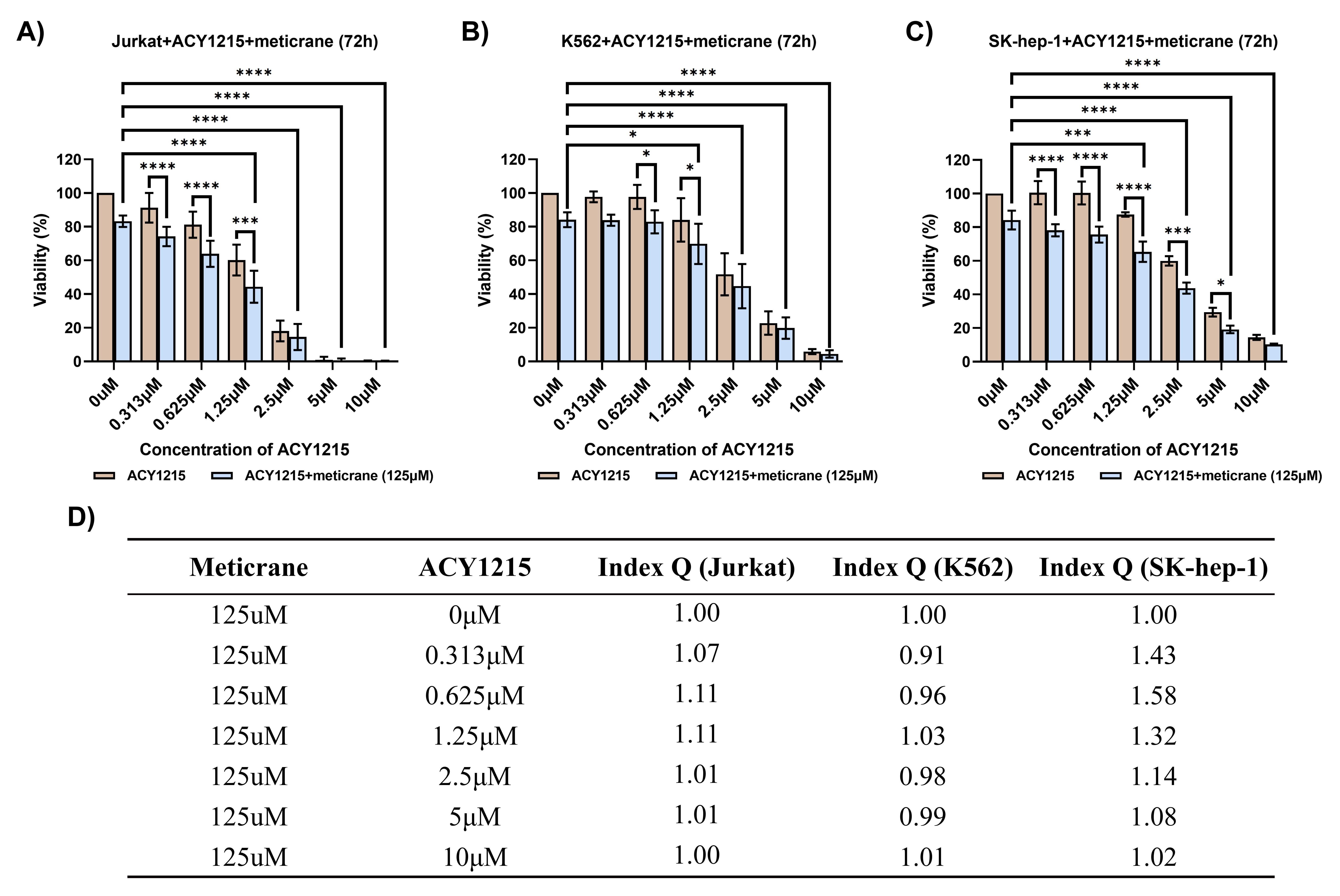

Supplement: Supplementary Figure 4 — The combination effect of meticrane with ACY1215 (selective HDAC6 inhibitor). The viability of Jurkat (A), K562 (B) and SK-hep-1 (C) cells in presence of ACY1215 with/without meticrane. D) Combination index Q of Meticrane and ACY1215 on K562, Jurkat and SK-hep-1 cells. [file Image_4.jpeg]

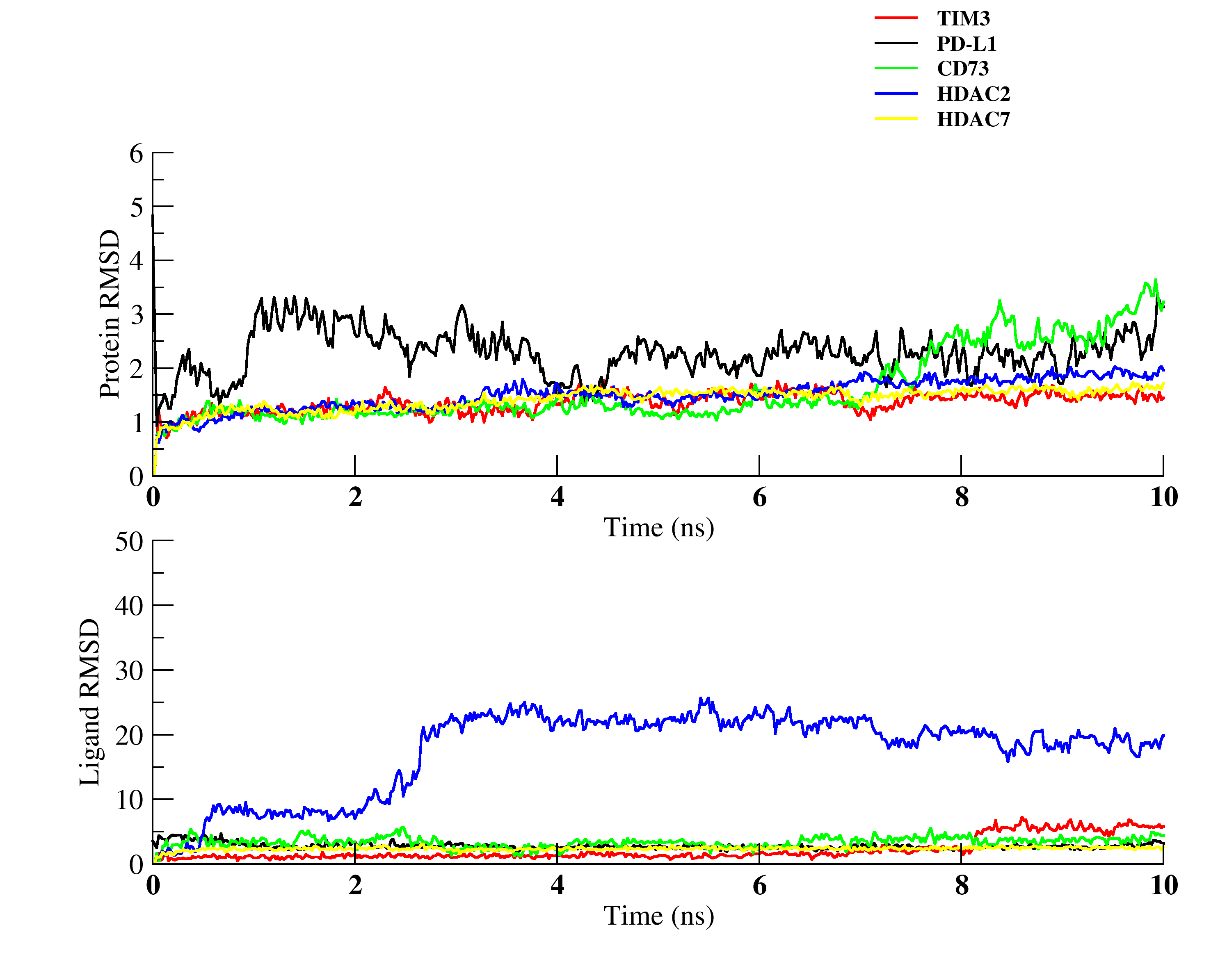

Supplement: Supplementary Figure 5 — Molecular dynamics [file Image_5.png]
